# Comparison of vonoprazan dual therapy, quadruple therapy and standard quadruple therapy for *Helicobacter pylori* infection in Hainan: a single-center, open-label, non-inferiority, randomized controlled trial

**DOI:** 10.1186/s12876-024-03225-8

**Published:** 2024-04-12

**Authors:** Chen Chen, Daya Zhang, Shimei Huang, Fan Zeng, Da Li, Xiaodong Zhang, Runxiang Chen, Shiju Chen, Jun Wang, Feihu Bai

**Affiliations:** 1 Graduate School, Hainan Medical University, Haikou, 571199 China; 2 Department of Gastroenterology, The 986 Hospital of Xijing Hospital, Air Force Military Medical University, Xi’an, 710054 Shanxi China; 3 Department of Gastroenterology, The Second Affiliated Hospital of Hainan Medical University, Yehai Avenue, #368, Longhua District, Haikou, 570216 Hainan Province China; 4 The Gastroenterology Clinical Medical Center of Hainan Province, Haikou, 570216 China

**Keywords:** *Helicobacter pylori*, Dual therapy, Quadruple therapy, Vonoprazan; non-inferiority

## Abstract

**Objective:**

To compare the potential efficacy and safety of dual therapy and quadruple therapy with vonoprazan (VPZ) as well as the standard quadruple therapy of proton pump inhibitor (PPI) for the eradication of *Helicobacter pylori* (Hp) infection in Hainan province.

**Methods:**

A single-centre, non-blinded, non-inferiority randomized controlled trial was conducted at the outpatient department of gastroenterology at the Second Affiliated Hospital of Hainan Medical University from June 2022 to February 2023. 135 patients aged 18–75 years with Hp infection were enrolled and randomized into three different groups (group V1: VPZ 20 mg twice a day and amoxicillin 1.0 g three times a day for 14 days V2: vonoprazan 20 mg, amoxicillin capsules 1.0 g, furazolidone 0.1 g and bismuth potassiulm citrate 240 mg, twice daily for 14 days;; group V3: ilaprazole 5 mg, Amoxicillin 1.0 g, Furazolidone 100 mg, bismuth potassiulm citrate 240 mg, twice a day for 14 days). Four weeks after the end of treatment, Hp eradication was confirmed by rechecking ^13^C-urea breath test (UBT).

**Results:**

The eradication efficacy of V1 and V3 was non-inferior to that of V2, which is consistent with the results obtained from the Kruskal-Wallis H test. The eradication rate by intentional analysis was 84.4% (38/45, 95%CI 73.4%–95.5%, *P*>0.05) for all the three groups. If analyzed by per-protocol, the eradication rates were 88.4% (38/43, 95%CI 78.4%–98.4%), 92.7% (38/41, 95%CI 84.4%–101.0%),88.4% (38/43，95%CI 78.4%–98.4%) in groups V1, V2 and V3, respectively, which did not show a significant difference (*P* > 0.05). The incidence of adverse effects was significantly lower in VPZ dual therapy compared to the other two treatment regimens (*P* < 0.05). VPZ dual therapy or quadruple therapy was also relatively less costly than standard quadruple therapy.

**Conclusion:**

VPZ dual therapy and quadruple therapy shows promise of not being worse than the standard quadruple therapy by a clinically relevant margin. More studies might be needed to definitively determine if the new therapy is equally effective or even superior.

**Supplementary Information:**

The online version contains supplementary material available at 10.1186/s12876-024-03225-8.

## Introduction

*Helicobacter pylori* (Hp) is a pathogen that primarily resides in the stomach and infects nearly half of the world’s population [1]. In China, the prevalence of Hp infection is approximately 40% [2]. Most people with Hp infection may not display any obvious gastrointestinal symptoms, or may only show mild indigestion symptoms. However, most infected individuals will have varying degrees of chronic gastritis, and about 15–20% of infected individuals might also develop peptic ulcers and, whereas in more severe cases, gastric cancer or mucosa-associated lymphoid tissue lymphoma [3]. According to the 5th National Consensus Report on the Management of Hp Infection in China, Hp eradication therapy has been recommended even for asymptomatic infected patients [4]. Hp is classified as a class I carcinogen by World Health Organization’s International Agency for Research on Cancer [5] and its eradication can serve as an important strategy to reduce the risk of cancer [6]. In China, quadruple therapy (Proton pump inhibitors(PPI) + two kinds of antibiotics + one bismuth agent) is the standard HP eradication treatment regimen [4], which generally has an eradication rate of over 80%, but can exhibit severe shortcomings, such as increasing antibiotic resistance and substantially higher adverse effects [7]. In China, Hp resistance to antibiotics such as clarithromycin (CLR), metronidazole and levofloxacin is continuously increasing [8] and is considered as the main reason for the decline in Hp eradication rates [9].

Successful eradication of Hp is associated with suppression of stomach acid in addition to antibiotic sensitivity [10, 11]. Vonoprazan(VPZ) is a new potassium competitive acid blocker (P-CAB) with strong acid-suppressive effects, which was first approved in Japan in 2015 for the treatment of Hp infection, reflux esophagitis and a range of other gastrointestinal diseases [12]. VPZ specifically acts on wall cells to bind to its H+/K+ ATPase transporter protein ions, thereby rapidly preventing K+ ions from entering the proton pump and substantially blocking H+ ions secretion, thus, acting as an acid suppressor [13]. VPZ can potentially inhibit 100% of gastric acid for 1 week, the average intragastric pH value can be then maintained at around 6.8, which has a stronger and longer-lasting acid-suppressive effect [14]. Eradication regimens with VPZ as an acid-suppressing drug exhibited higher eradication rates than PPI eradication regimens [15–17]. Therefore, this paper aimed to compare the efficacy and safety of VPZ’s dual therapy with VPZ’s quadruple therapy as well as standard PPI’s therapy for Hp infection eradication in Hainan province.

## Patients and methods

### Patients and study design

This randomized controlled trial was conducted from June 2022 to February 2023 in the outpatient clinic of the Department of Gastroenterology, Second Affiliated Hospital of Hainan Medical University. Ethics committee approval was obtained for the conduct of the study. The inclusion criteria used were: (1) 13C urea breath test positive confirmed diagnosis of *H. pylori* infection in patients who are 18–75 years of age;(2) none of these infected patients had any previous history of diseases such as severe liver insufficiency, renal insufficiency, gastrointestinal tumor, and history of PPI, antibiotic, or other drug use within the past 4 weeks; (3) all patients agreed to participate in the study and signed an informed consent form. Exclusion criteria included were (1) history of gastrointestinal tumors or surgery, (2) allergy to any of the drugs used in the study, (3) patients with severe mental disorders that prevented communication, (4) refusal to participate in the study. Ultimately 135 Hp-infected patients were included in the study.

This was a randomized controlled trial in which study participants were numbered in order of visit, 1–135. The treatment regimen that each subject receives is generated from the resulting random allocation sequence generated by an investigator not involved in data collection using Research Randomizer software (http://www.randomizer.org) and is placed in a sequential, sealed, opaque envelope. The envelope is opened and the subject receives the appropriate treatment when an eligible subject agrees to enter the trial. Study participants were randomly assigned to the three treatment groups according to a 1:1:1 ratio. During the design of the study, we took into account that the knowledge of the complete treatment regimen by the study subjects might be beneficial to increase the compliance and cooperation of the subjects, which would facilitate the conduct of the study and the recruitment of the subjects. Therefore, subjects were aware of their subgroups and the medications they were taking. In the course of this clinical study, the observation of indicators, the collection of data and the formation of conclusions were carried out without knowledge of the group in which the subjects were placed and without knowledge of the type of measure received.

This clinical trial study was reviewed and approved by the Ethics Committee of the Second Affiliated Hospital of Hainan Medical University (No. LW2023113). The trial was registrated on 17/10/2023 (ChiCTR2300076723).

### Sample size calculation

Based on the expert consensus on statistical considerations in the design of non-inferiority clinical trials developed by the China Clinical Trial Statistical Study Group (CCTS) and the guidelines for the design of non-inferiority trials developed by the FDA, we developed non-inferiority cut-offs for the trials based on the degree of net benefit of efficacy in the control group. According to previous similar studies, the eradication rate was 98.5% for vonoprazan dual therapy [18], 97.4% for vonoprazan quadruple therapy and 96.7% for conventional PPI [19, 20]. The study was a non-inferiority study with the non-inferiority value set at 10, 80% certainty (Power is 0.8), and a test level of 0.05 (unilateral), assuming the same sample size for each group. PASS 15.0 was used for sample size calculation. The results showed that at least 19 people/group were needed based on the V1 eradication rate, at least 32 people/group were needed based on the eradication rate in group V2, and at least 40 people/group were needed based on the eradication rate in group V3. Taking the maximum value from the calculations, a minimum of 40 people were needed in each group, and a total of 120 people were needed in the three groups, and taking into account that there may be a 10% lost visit rate, it was finally decided to include 135 people (Sample size calculation using pass 15).

### Treatments and follow-up

135 patients were randomly assigned to one of three different eradication regimens. Group V1: The patients were administered vonoprazan 20 mg twice daily and amoxicillin (AMX) capsules 1.0 g three times daily for 14 days; Group V2: The patients were administered vonoprazan 20 mg, amoxicillin capsules 1.0 g, furazolidone 0.1 g and bismuth potassiulm citrate 240 mg, twice daily for 14 days; Group V3: The patients were administered ilaprazole 5 mg, Amoxicillin 1.0 g, Furazolidone 0.1 g and bismuth potassiulm citrate 240 mg twice daily for a fortnight. Acid suppressants and bismuth were administered half an hour before the meals and antibiotics were given half an hour after the meals [4]. After 14 days of continuous administration, medication was discontinued for 1 month and a ^13^C urea breath test to determine the eradication status of Hp. Overall considering the interests of the participants, we selected to sue amoxicillin and furazolidone, which have low resistance rates in China. It has been reported that compared to these two antibiotics, CLR and metronidazole have higher resistance. The doses of antibiotics used in the quadruple regimen are those recommended in the national consensus [4]. The dosage was increased after considering that there was only one antibiotic included in the dual therapy. The patients were followed up by telephone during and after the medication to determine whether the medication was administered regularly and whether any other adverse reactions developed during the course of administration.

### Study outcome

The primary study endpoint was the eradication rate for Hp infection of the three treatment regimens, with secondary study endpoints of safety and economic benefit. A successful eradication was considered to be achieved if the ^13^C-UBT showed a DOB < 4.0 after 14 days of regulated dosing and 1 month of drug withdrawal. However, in the per-intent analysis, patients lost to the -up and those who failed to take the medication regularly for 14 days for multiple reasons were considered for the failed eradication in enrolled patients. We considered the treatment options to have an acceptable eradication rate when the eradication rate was > 85%.

A total cost in Group V1 is $ 57.46 (vonoprazan 28 tablets were $40.63 and amoxicillin 168 capsules were $16.83). A total cost in Group V2 was $60.58 (vonoprazan(20 mg/capsule) 28 tablets were $40.63, bismuth potassiulm citrate (120 mg/capsule) 56 capsules were $8.13, amoxicillin (250 mg/capsule)112 capsules were $11.21 and furazolidone(100 mg/capsule) 28 capsules were $0.61). A total cost in Group V3 was $ 70.27 (ilaprazole(5 mg/capsule)28 tablets were $50.32, bismuth potassiulm citrate 56 capsules were $8.13, amoxicillin 112 capsules were $11.21 and furazolidone 28 capsules were $0.61). The cost divided by the eradication rate can yield cost effectiveness ratio (CER) for the three different treatment options. A higher CER indicates that the treatment option was less cost effective.

### Statistical analysis

Categorical variables were expressed as ratios, and differences between groups were analyzed by the Kruskal-Wallis H test and multiple corrections were performed using the Bonferroni test. Continuous variables were expressed as mean (±) standard deviation, and comparisons were made using analysis of variance (ANOVA) under conditions consistent with normality. SPSS 26 was used to complete the above analysis. In addition, non-inferiority was tested using SAS 9.4, with the non-inferiority value set at 10%. Non-inferiority was established if the lower limit of the 95% confidence interval for the difference in eradication rates was > − 10%.

## Results

**Fig. 1:**
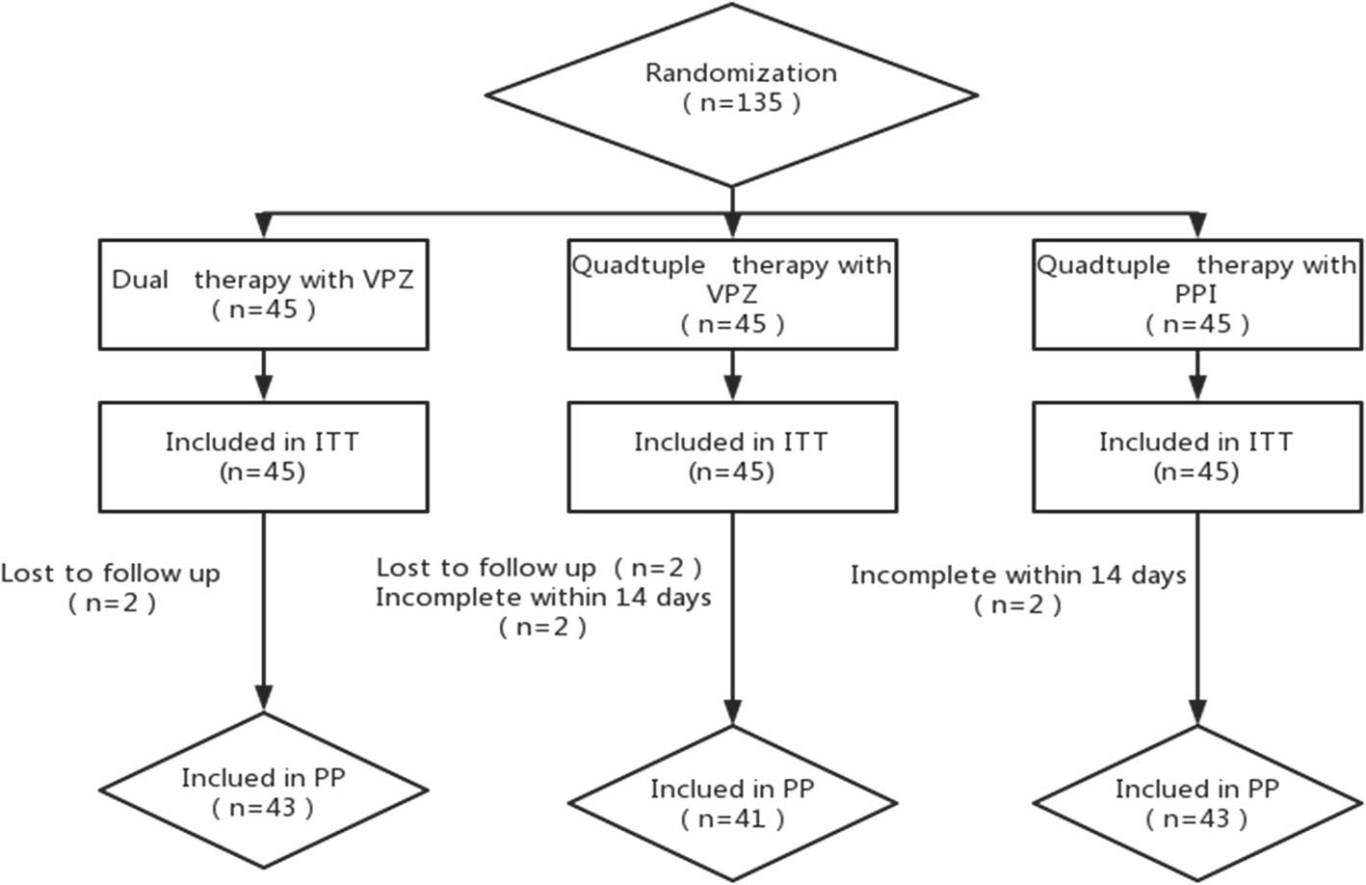
Flow chart of the randomized grouping of patients enrolled in the study

We found that 2 patients were lost to follow-up and 43 eventually completed treatment in Group V1. 2 were lost to follow-up, 2 were discontinued due to various intolerable adverse effects, and 41 eventually completed treatment in Group V2. 2 patients discontinued the therapy due to intolerable adverse effects and 43 eventually completed treatment in Group V3 (depicted in Fig. 1). Table 1 summarizes the demographic clinical characteristics of enrolled patients. There were no statistically significant differences observed in demographic characteristics between the three groups of patients on different treatment regimens.

**Table 1:** Demographic characteristics of the patients enrolled in the study

Variables	V1 (*n* = 45)	V2 (*n* = 45)	V3 (*n* = 45)	*P* Value	H/F
Gender					
Male	20 (44.4%)	22 (48.9%)	25 (55.6%)	0.572	1.118
Female	25 (55.6%)	23 (51.1%)	20 (44.4%)		
Age (mean, SD,y)	44.87 ± 2.00	42.44 ± 1.83	40.78 ± 1.49	0.269	1.325
Range Living area					
Urban	22 (48.9%)	24 (53.3%)	26 (57.8%)	0.702	0.709
Villages and towns	23 (51.1%)	21 (46.7%)	19 (42.2%)		
High	17 (37.8%)	20 (44.4%)	16 (35.6%)	0.670	0.802
Low	28 (62.2%)	25 (55.6%)	29 (64.4%)		
Dining Style					
Eat at home	38 (84.4%)	37 (82.2%)	33 (73.3%)	0.381	1.930
Dining out	7 (15.6%)	8 (17.8%)	12 (26.7%)		
Communal tableware					
Frequently	30 (66.7%)	32 (71.1%)	31 (68.9%)	0.902	0.206
Rarely	15 (33.3%)	13(28.9%)	14 (31.1%)		

**Fig. 2:**
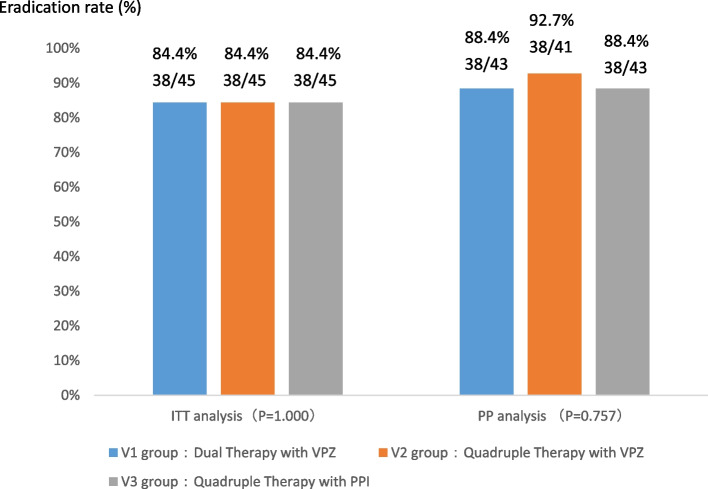
Comparison of the Hp eradication rates in three different treatment regimens

According to the findings of the multiple non-inferiority test, the lower limit of the 95%CI for the difference in eradication rates between the two groups (V1 vs V2) and the two groups (V3 vs V2) in the ITT analysis was less than the non-inferiority value, and it did not conform to non-inferiority. However, in the PP analysis, the lower limit of the 95% CI for the difference in eradication rates between the two groups and the two groups was greater than the non-inferiority value. That is, according to the actual eradication rates, the eradication efficacy of V1 and V3 was non-inferior to that of V2, which is consistent with the results obtained from the Kruskal-Wallis H Test (Tables 2–3). Hp was eradicated in 38 patients in group V1, with an eradication rate of 84.4% (38/45, 95%CI 73.4–95.5%) by ITT analysis and an eradication rate of 88.4% (38/43, 95%CI 78.4–98.4%) by PP analysis. Moreover Hp was eradicated in 38 patients in group V2, with an ITT eradication rate of 84.4% (38/45, 95%CI 73.4–95.5%) by ITT analysis and an eradication rate of 92.7% (38/41, 95%CI 84.4–101.0%) by PP analysis. Finally, Hp was also eradicated in 38 patients in group V3, with an eradication rate of 84.4% (38/45, 95%CI 73.4–95.5% by ITT analysis and an eradication rate of 88.4% (38/43, 95%CI 78.4–98.4%) by PP analysis. There was no significant difference in the Hp eradication rate between the three groups (*p* > 0.05) (Fig. 2 and Table 3). This study involved three subgroups, and in the multiple hypothesis correction test (Table S1), there was no statistical difference in the findings between these subgroups V1, V2, and V3.

**Table 2:** Multiple non-inferiority analysis for Hp eradication rates

	Group	Eradication rate And 95% CI (ITT)	Eradication rate And 95% CI (PP)	Difference [95% CI] (ITT)	Difference [95% CI] (PP)	*P* ^†^
V1 and V2	V1	84.4% 73.4–95.5%	88.4% 78.4–98.4%	−14.9-14.9%	−8.2-16.8%	0.095(ITT) 0.186(PP)
	V2	84.4% 73.4–95.5%	92.7% 84.4–101.0%			
V3 AndV2	V3	84.4% 73.4–95.5%	88.4% 78.4–98.4%	−14.9-14.9%	−8.2-16.8%	0.095(ITT) 0.186 (PP)
	V2	84.4% 73.4–95.5%	92.7% 84.4–101.0%			

*P* ^†^ *p*-value for non-inferiority test

*CI* confidence interval, *ITT* intention-to-treat, *PP* per-protocol

**Table 3:** Kruskal-Wallis H Test analysis for Hp eradication rates

	Group	Eradication rate	*P*	H(K)
ITT	V1	38/45 (84.4%)	1.000	0.000
	V2	38/45 (84.4%)		
	V3	38/45 (84.4%)		
PP	V1	38/43 (88.4%)	0.757	0.557
	V2	38/41 (92.7%)		
	V3	38/43 (88.4%)		

*P p*-value for Kruskal-Wallis H Test

*ITT* intention-to-treat, *PP* per-protocol

The most common adverse reaction to standard quadruple therapy was abdominal pain (Table 4). The incidence of nausea was markedly higher in group V2 in comparison to the other two treatment regimens (*P* < 0.05). The incidence of adverse reactions in groups V1, V2 and V3 were 2.3% (1/43), 19.5% (8/41) and 14.0% (6/43) respectively. The incidence of adverse reactions in groups V1 was significantly less in comparison to the other two treatment regimens (*p* < 0.05). In the multiple hypothesis correction test (Table S2), there was no statistical difference in the nausea between these subgroups V1, V2, and V3. While V1 differ V2 in the incidence of adverse reactions.

**Table 4:** Comparison of major adverse events between the three treatment options

	Group-V1 (*n* = 43)	Group-V2 (*n* = 41)	Group-V3 (*n* = 43)	*P* Value	H
Overall	1 (2.3%)	8 (19.5%)	6 (14.0%)	< 0.05	6.189
Skin eruption	0	1 (2.4%)	0	0.350	2.098
Nausea	1 (2.3%)	6 (14.6%)	1 (2.3%)	< 0.05	7.070
Dizziness	1 (2.3%)	2 (4.9%)	1 (2.3%)	0.745	0.588
Inappetence	0	1 (2.4%)	0	0.350	2.098
Abdominal pain	0	0	3 (7.0%)	0.051	5.955
Abdominal bloating	0	0	1 (2.3%)	0.377	1.953
Constipation	0	0	1 (2.3%)	0.377	1.953
Easy fatigability	0	1 (2.4%)	0	0.350	2.098

The V3 group incurred the highest medical costs and cost-benefit ratios (Table 5). The V1 and V2 regimen incurred lower medical costs, lower cost-effectiveness ratios as well as higher economic benefits than the V3 regimen.

**Table 5:** Cost-effectiveness ratios

	Group	Total cost	Eradication rates	CER
ITT	V1	57.46	84.4%	0.68
	V2	60.58	84.4%	0.72
	V3	70.27	84.4%	0.83
PP	V1	57.46	88.4%	0.65
	V2	60.58	92.7%	0.65
	V3	70.27	88.4%	0.79

## Disscussion

The success rate of HP eradication regimens involving PPIs is continuously decreasing due to increased antibiotic resistance as well as their insufficient acid-suppressive effect,.Therefore, it is difficult to improve the therapeutic efficacy of PPIs even when their dosage is increased [21]. VPZ can effectively control the gastric PH above 4.0 most of the time with a dose of 20 mg twice daily, which can exhibit as strong and a long period acid-suppressing effect [22–24]. we observed that patients with successful Hp eradication displayed a higher range of fluctuation in 24-hour gastric pH than those with failed eradication, and a higher pH has been reported to be beneficial for Hp eradication [25]. Amoxicillin and clarithromycin, which are commonly used in Hp eradication, showed the best antibacterial effect at a pH of 6–7 [26].

As indicated in Table 6, our results are similar to the recent findings of Lifen Lu et al. who reported that VPZ quadruple therapy, whether administered for 10 days or 14 days, can achieve eradication rates higher than 90% and was not inferior to standard PPI quadruple therapy [19]. VPZ has been included as an acid-suppressing drug in first-line and second-line Hp eradication regimens and the secondary eradication rate was found to be above 90% in Japan [34]. Interestingly, in a study by YI Hu and Wen Gao, the eradication rate of high-dose AMX in combination with VPZ was also observed to be above 85% [27, 28], but some studies have shown that the eradication rate of VPZ dual therapy was not satisfactory [7]. According to PP analysis, the VPZ dual therapy in this study demonstrated an acceptable eradication effect (eradication rate > 85%). Perhaps enhanced acid suppression and prolonged treatment duration are beneficial in improving the eradication rate of VPZ dual therapy[7，30–31]. Consider quadruple therapy if dual therapy is controversial.due to，In studies of VPZ quadruple therapy, Hp eradication rate mostly exceeded 90% [19, 29, 30]. In addition to dual and quadruple therapies, the VPZ triple therapy in foreign studies also seems to have good eradication results, but prolonged treatment time does not show an advantage in triple therapy [33].

**Table 6:** Vonoprazan-related studies in recent years

Year and Author	Treatment options	Sample size	Eradication rate		*P* Value
			ITT	PP	
2022 Yimin Lin [7]	I: VPZ 20 mg bid+AMX 750 mg qid (7 days) II: VPZ 20 mg bid+AMX 500 mg qid (7 days) III:VPZ 20 mg bid+AMX 750 mg bid+clarithromycin 500 mg (7 days)	230	63.5% 58.3% 60.7%	65.1% 66.2% 64.9%	*P* > 0.05
2022 Lifen Lu [19]	V10:VPZ20mg + AMX1.0 g + Furazolidone 100 mg + colloidal bismuth 200 mg (bid 10 days) V14:VPZ20mg + AMX1.0 g + Furazolidone 100 mg + colloidal bismuth 200 mg (bid 10 days) E14: esomilaprazole 20 mg + AMX 1.0 g + Furazolidone100 mg + colloidal bismuth 200 mg (bid 14 days)	234	V10: 96.2% V14:94.9% E14:93.6%	V10:98.6% V14:97.4% E14:94.8%	*P* > 0.05
2022 YI Hu [27]	VPZ 20 mg bid+AMX1.0 g Bid (7 days) VPZ20mg bid+AMX1.0 g Bid (10 days) VPZ 20 mg bid+AMX1.0 g tid (7 days) VPZ 20 mg bid+AMX1.0 g tid (10 days)	119	66.7% 89.2% 81.0% 81.1%	72.7% 89.2% 81.0% 81.1%	*P* > 0.05
2022 Wen Gao [28]	VPZ 20 mg/40 mg + AMX1.0 g tid (14 days)	186	92.5%		–
2023 Juan Wang [29]	VPZ 20 mg + AMX 1.0 g + CLR 500 mg + colloidal bismuth 200 mg (bid 14 days) lansoprazole 30 mg / esomilaprazole 20 mg + AMX 1.0 g + CLR 500 mg + colloidal bismuth 200 mg (bid 14 days)	340	88.8% 87.6%	94.1% 91.1%	*P < *0.01
2021 Huh [30]	VPZ 20 mg + AMX 1.0 g + CLR 500 mg + colloidal bismuth 200 mg (bid 14 days) Lansoprazole 30 mg + AMX 1.0 g + CLR500mg + colloidal bismuth 200 mg (bid 14 days)	36	100% 100%	100% 100%	*P* > 0.05
2020 Sho Suzuki [31]	VPZ 20 mg tid + AMX 750 mg tid (7 days) VPZ 20 mg tid + AMX 750 mg tid. + clarithromycin 200 mg tid(7 days)	335	84.5% 89.2%	87.1% 90.2%	*P* > 0.05
2020 William D Chey [32]	VPZ 20 mg bid+ AMX 1000 mg tid (14 days) VPZ 20 mg bid + AMX 1000 mg bid. + clarithromycin 500 mg bid(14 days) lansoprazole 30 mg bid + AMX 100 mg bid. + clarithromycin 500 mg bid(14 days)	1046	78.5% 84.7% 78.8%	81.2% 90.4% 82.1%	*P* < 0.01 (ITT) *P* = 0.016(PP)
2021 Chalermrat Bunchorntavakul [33]	VPZ 20 mg bid+ AMX 1000 mg bid. + clarithromycin 500 mg bid(7 days) VPZ 20 mg bid+ AMX 1000 mg bid. + clarithromycin 500 mg bid(14 days)	122	96.7% 88.5%	98.3% 93.1%	*P* > 0.05

In terms of adverse reactions, adverse reactions observed in the VPZ regimen were mainly dizziness and nausea, especially nausea was apparently more frequent in VPZ quadruple therapy, whereas major adverse reaction in standard PPI therapy group was pronounced abdominal pain. Additionally, in previously reported studies on quadruple therapy, although a high eradication rate was achieved, the incidence of adverse reactions was relatively as high as 30% for VPZ quadruple therapy and 27.1% for standard PPI quadruple therapy [29]. Moreover, in addition to common GI adverse effects, HP regimens containing VPZ have been reported to cause systemic reactions such as chest pain, generalized pain and dizziness [35]. The higher incidence of adverse reactions associated with VPZ quadruple therapy and standard therapy can significantly affect patients’ compliance and quality of life during the treatment, which should be taken into consideration. Therefore, VPZ dual therapy can be a good choice for the treatment of Hp infection.

Our study has some limitations: (1) It was a single-centre randomized controlled trial and the sample size was small, further multi-centre, large sample studies are still needed to validate our results; (2) This study did not differentiate between patients who had been initially eradicated of Hp and those who were re-eradicated of Hp, and HP infections were not cultured in these study participants, lacking data on HP drug resistance. Drug resistance may adversely affect eradication rates and influence study results; (3) participants were not monitored for 24 hours for PH, which may also adversely affect eradication rates.

In conclusion, VPZ dual therapy and quadruple therapy shows promise of not being worse than the standard quadruple therapy by a clinically relevant margin. More studies might be needed to definitively determine if the new therapy is equally effective or even superior. In other aspects, VPZ dual therapy is safer, less economically costly.

### Supplementary Information

**Supplementary Material 1.**

## Data Availability

The datasets generated and/or analyzed during the current study are available from the corresponding author upon reasonable request.

## Abbreviations

*H. pylori*: *Helicobacter pylori*
VPZ: Vonoprazan
PPI: Proton pump inhibitor
UBT: Urea breath test
CLR: Clarithromycin
AMX: Amoxicillin
AEs: Adverse events
MPR: Medication possession rate
ITT: Intention-to-treat
PP: Per-protocol
CI: Confidence interval
MIC: Minimum inhibitory concentration
