# Supplementary material for: Comparison of vonoprazan dual therapy, quadruple therapy and standard quadruple therapy for Helicobacter pylori infection in Hainan: a single-center, open-label, non-inferiority, randomized controlled trial
Source: BMC Gastroenterol. 2024 Apr 12;24:131. doi: 10.1186/s12876-024-03225-8 (PMC11010374; doi:10.1186/s12876-024-03225-8)
Supplement: Supplementary file 1 — Supplementary Material 1. [file 12876_2024_3225_MOESM1_ESM.docx]

Table S1. Multiple testing Hp eradication rates by corrections (Bonferroni or Benjamini-Hochberg)

|  |  | V1 | V2 | V3 | total |
| --- | --- | --- | --- | --- | --- |
| Eradication success | count（ITT） | 38_a_ | 38_a_ | 38_a_ | 114 |
|  | ratio（ITT） | 84.4% | 84.4% | 84.4% | 84.4% |
|  | count（PP） | 38_a_ | 38_a_ | 38_a_ | 114 |
|  | ratio（PP） | 88.4% | 92.7% | 88.4% | 89.8% |
| Eradication failure | count（ITT） | 7_a_ | 7_a_ | 7_a_ | 21 |
|  | ratio（ITT） | 15.6% | 15.6% | 15.6% | 15.6% |
|  | count（PP） | 5_a_ | 3_a_ | 5_a_ | 13 |
|  | ratio（PP） | 11.6% | 7.3% | 11.6% | 10.2% |
| total | count（ITT） | 45 | 45 | 45 | 135 |
|  | ratio（ITT） | 100.0% | 100.0% | 100.0% | 100.0% |
|  | count（PP） | 43 | 41 | 43 | 127 |
|  | ratio（PP） | 100.0% | 100.0% | 100.0% | 100.0% |

The subscript letters all indicate subsets of the grouped categories, which are not significantly different from each other at the calibration level α = 0.05.

Table S2. Multiple testing Incidence of adverse reactions by corrections (Bonferroni or Benjamini-Hochberg)

|  |  |  | V1 | V2 | V3 | total |
| --- | --- | --- | --- | --- | --- | --- |
| Overall incidence of adverse reactions | Yes | count | 42_a_ | 33_b_ | 37_a, b_ | 112 |
|  |  | ratio | 97.7% | 80.5% | 86.0% | 88.2% |
|  | NO | count | 1_a_ | 8_b_ | 6_a, b_ | 15 |
|  |  | ratio | 2.3% | 19.5% | 14.0% | 11.8% |
|  | Total | count | 43 | 41 | 43 | 127 |
|  |  | ratio | 100.0% | 100.0% | 100.0% | 100.0% |
| Incidence of nausea | Yes | count | 42_a_ | 35_a_ | 42_a_ | 119 |
|  |  | ratio | 97.7% | 85.4% | 97.7% | 93.7% |
|  | NO | count | 1_a_ | 6_a_ | 1_a_ | 8 |
|  |  | ratio | 2.3% | 14.6% | 2.3% | 6.3% |
|  | Total | count | 43 | 41 | 43 | 127 |
|  |  | ratio | 100.0% | 100.0% | 100.0% | 100.0% |

The subscript letters all indicate subsets of the grouped categories, which are not significantly different from each other at the calibration level α = 0.05.
